# Rosin Solution for Root Filling

**Published:** 1914-06-15

**Authors:** 


ROSIN SOLUTION FOR ROOT FILLING. Dr. J. R. Callahan has had his article on the use of "Rosin Solution for the sealing of the Dental Tubuli and as an adjuvant in the filling of root canals" reprinted in pamphlet form from the Journal of Allied Sciences. The text and illustrations make the technique clear and readily comprehensible. This method of root canal work should do much to make this operation definite and assure better work with less probability of septic consequences. Every dentist should have a copy of this reprint.

